# Announcment: Joint IBDG/SBIC Meeting Metals in Biology: From Homeostasis to Medical Applications

**DOI:** 10.1155/MBD.1995.337

**Published:** 1995


					Joint IBDG/SBIC MEETING
Metals in Biology:
from Homeostasis to Medical Applications
To be held at The "Universit Catholique de Louvain-la-Neuve",
Louvain-la-Neuve, Belgium, 16th May-19th May 1996
Thursday 16th May 1996
17.00 Calcium Homeostasis
Professor E. Carafoli, Zerich, Switzerland
18.00 WELCOME RECEPTION
Friday 17th May 1996
09.00 Iron Homeostasis in Bacteria
Professor V Braun, TObingen, Germany
10.00 COFFEE and Poster Viewing
11.00 Translational Regulation of Iron Metabolism in Animal Cells
Dr L Kuhn, Epalinges, Switzerland
13.00 LUNCH
14.00 Copper Homeostasis in Bacteria
Professor NL Brown, Birmingham, UK
15.00 Copper Homeostasis in Man
Professor HJ McArdle
16.00 Poster Viewing
17.00 Poster Presentations
18.001 Iron and Copper Interactions
Speaker to be confirmed
Saturday 18th May 1996
09.00 Contrast Agents in MRI
Professor Bertini, Firenze, Italy
10.00 COFFEE
10.30 Long-lived Platinum-DNA Monoadducts: Statergy for DNA- Protein Cross-linking
Dr J-C Chottard, Paris, France
11.30 Young scientist Presentation
12.30 LUNCH
14.00 Entitumor Metallocenes New Experimental and Clinical Results
Professor P. K6pf-Maier, Berlin, Germany
15.00 COFFEE and Poster Viewing
16.00 Poster Presentations
17.00 Metallodrugs: Transport and Metabolism
Professor P Sadler, London, Britain
19.00 CONFERENCE DINNER
For further information, please contact:
Prof. R. R. Crichton
UCL, CHIM/BIOC, B&timent Lavoisier, Place Pasteur
B-1348 Louvain-la-Neuve, Belgium
E-mail: CRICHTON@ BIOC.UCL.AC.BE
Fax: + 32 10-472 796
Phone: + 32 10 472794 and 2796)
337

				

